# Bioenhancing effects of naringin on atorvastatin

**DOI:** 10.5599/admet.647

**Published:** 2019-06-06

**Authors:** Venkatesh Sama, Balaraju Pagilla, Rajeswari Chiluka, Ravi Alvala, Ravi Kumar Pola, Ramesh Mullangi

**Affiliations:** 1 G. Pulla Reddy College of Pharmacy, Mehdipatnam, Hyderabad-500028, India; 2 JubilantBiosys, Industrial Suburb, Yeshwanthpur, Bangalore-566022, India

**Keywords:** Naringin, Bioenhancer, Atorvastatin, Tyloxapol, Plasma concentrations, RP-HPLC

## Abstract

Naringin (CAS no: 10236-47-2) is a flavonone glycoside obtained from Citrus paradisi (grapefruit), a natural bioenhancer and reported to enhance the bioavailability of drugs by inhibiting cytochrome P450 and P-glycoprotein (P-gp). The aim of the present study was to investigate the effect of naringin on antihyperlipidemic properties of atorvastatin (AST) in tyloxapol induced hyperlipidemic rats and the effects were supported with measurement of plasma concentrations of AST by HPLC method. Animals received AST along with naringin (15 and 30 mg/kg) shown higher percent reduction in both cholesterol and triglycerides levels, when compared to animals received AST alone at dose of 25 and 50 mg/kg and it was found that the higher percent reduction in cholesterol and triglycerides was proportional to increase in plasma concentration of AST. From the results it is evident that the co-administration of naringin along with AST increased the plasma concentration of AST. The findings of the present study confirmed that naringin could be used as bioenhancer. The co-administration of AST and the diet with naringin (grapefruit) to the patients may potentiate the therapeutic efficacy of AST.

## Introduction

Recent advancement in bioavailability enhancement of drugs by compounds of herbal origin has produced a revolutionary shift in the way of therapeutics. Poorly bioavailable drugs remain sub-therapeutic because a major portion of a dose never reaches the plasma or exerts its pharmacological effect unless and until very large doses are given, which may lead to serious side effects. Any significant improvement in bioavailability will result in lowering the dose or the dose frequency of that particular drug. The global focus is now on methods aimed at reducing drug dosage, and thus drug treatment cost [1]. Bioenhancers of herbal origin are reported to enhance the bioavailability and bioefficacy of a therapeutic drugs or nutrients with which it is combined, without any typical pharmacological activity of its own at the dose used. The concept of bioenhancers of herbal origin can be tracked back from the ancient knowledge of Indian system of medicine (Ayurveda). Bose (1929) reported an enhanced antiasthmatic effect of an Ayurvedic formula containing vasaka (Adhatoda vasica) when administered with long pepper [2]. Bioenhancers act through several mechanisms. They affect the absorption process, drug metabolism or on drug target. Among the known reasons for the use of bioenhancers is its non-toxicity, effective at low concentration levels and simple formulation processes. Bioenhancers are effective when administered with other drug classes such as antibiotics, antituberculosis, antiviral, antifungal and anticancer drugs. Bioenhancers also improve oral absorption of a wide range of nutrients such as vitamins, minerals and amino acids etc. [3].

Naringin (7-[[2-O-(6-deoxy-α-L-mannopyranosyl)-β-D-glucopyranosyl]oxy]-2,3-dihydro-5-hydr- oxy-2-(4-hydroxyphenyl)-4H-1-benzopyran-4-one), a flavanone glycoside (rutinoside), occurs naturally in the pericarp of citrus fruits and grapefruits (Citrus paradisi; Family: Rutaceae) [4]. Naringin possess the ability of inhibition of the P-gp efflux pump [5,6] and also inhibit cytochrome P450 in particular CYP3A4 [7-9]. Literature search revealed that naringin has antiviral 10], anticancer [11], hepatoprotective [12], anti-inflammatory [13], anti-ulcer [14,15] and antioxidant activities [16]. Naringin has an inhibitory effect against CYP3A4 activity in human liver microsomes [9] and promotes ascorbic acid induced lipid peroxidation [17]. Naringin has a potent acyl CoA-cholesterol-O-acyltransferase (ACAT-promotes the esterification of cholesterol in blood) inhibitory activity, macrophage-lipid complex accumulation inhibitory activity and preventive or treating activity on the hepatic diseases [18]. Previous studies have reported that pretreatment of naringin appeared to be effective to alter the pharmacokinetics of drugs (verapamil, diltiazem, paclitaxel, tamoxifen) that are substrates of P-gp and/or CYP3A over the dose range of 3-30 mg/kg in rats/rabbits [19-22].

Atorvastatin (AST) chemically is a calcium salt of (βR, δR)-2-(4-fluorophenyl)-β,δ-dihydroxy-5-(1-methylethyl)–3-phenyl-4-[(phenylamino)-carbonyl]-1H-pyrrole-1-heptanoic acid (2:1) trihydrate. AST (Lipitor®) is a 3-hydroxy-3-methyl-glutaryl coenzyme A (HMG-CoA) reductase inhibitor and used for treating various dyslipidemic disorders. AST is rapidly absorbed from gastrointestinal tract. The extent of absorption increases in proportion to the dose and the absolute bioavailability is 12 %. Protein binding is very high (≥98 %) and undergoes extensive hepatic or extra hepatic metabolism. Pharmacokinetic studies of AST revealed the formation of ortho and parahydroxylated derivatives as primary metabolites. The cytochrome P450 family in particular CYP3A4 is potential to catalyse the formation of ortho and parahydroxylated derivatives [23,24]. The wide range of doses ranging from 10-80 mg of AST is in use as per the lipid lowering profile requirement. Due to low bioavailability of AST high dosing and/or repeated administration is required to achieve steady state of *C*_max_.

This research paper aims at reporting the effect of naringin on the antihyperlipidemic activity of AST in tyloxapol induced hyperlipidemia in Wistar rats. The observed activity was correlated with plasma concentration of AST in experimental animals.

## Materials and methods

### Chemicals and reagents

A gift sample of AST (purity >99.5 %) is obtained from M/S, Biocon Pharmaceuticals, Bangalore, India. Naringin procured from Sigma-Aldrich, Mumbai, India. Tyloxapol (isooctyl-polyoxyethylene phenol/Triton WR 1339) was purchased from Himedia (B. No: 25301-02-4) Mumbai, India. HPLC grade water, acetonitrile, methanol and orthophosphoric acid were purchased from SD Fine-Chem Limited and sodium dihydrogen orthophosphate from Otto Chemicals, Mumbai, India. Disodium salt of EDTA is procured from SD Fine-Chem Limited, Mumbai, India. Anesthetic ether is obtained from TKM Pharma, Hyderabad, India. Diagnostic kits for total cholesterol and triglycerides were obtained from Span Diagnostics Ltd, Surat, Gujarat, India. Cellulose acetate filters, pore size 0.2 μm obtained from Sartorius Stedim Biotech, Germany. All aqueous solutions including the buffer for mobile phase was prepared with HPLC grade water. Control Wistar rat plasma was obtained from Department of Pharmacology G. Pulla Reddy College of Pharmacy, Hyderabad, India.

### Experimental animals

Male Wistar rats weighing (~3 months age, weighing 180-200 g) were procured from National Institute of Nutrition, Hyderabad, India. Animals were maintained in standard cages under controlled laboratory conditions. The animals had free access to feed (National Institute of Nutrition, Hyderabad) and water ad libitium during quarantine period of seven days. Animals were fasted ~12 h before experiment but had been allowed free access to water. The Institutional Animal Ethics Committee of G. Pulla Reddy College of Pharmacy, Hyderabad, India has approved the animal experimental protocols.

### Effect of naringin on AST in tyloxapol induced hyperlipidemia

The method tyloxapol induced hyperlipidemia in rats was performed as described by Vogel [25]. Hyperlipidemia was induced by single intraperitonial injection of 15 % w/v tyloxapol in sterile normal saline at a dose of 400 mg/kg. After 30 h, rats with marked hypercholesterol were separated and divided into 10 groups of 6 animals each. All the test substances were administered orally as a fine aqueous suspension of 0.5 % w/v carboxy methyl cellulose (CMC). Group 1 served as disease control and received vehicle. Group 2 and 3 received naringin at a dose of 15 and 30 mg/kg, respectively. Animals of Group 4 received AST at a dose of 25 mg/kg; Group 5 and 6 received AST along with naringin at 25 & 15 and 25 & 30 mg/kg, respectively. Group 7 received AST 50 mg/kg; animals of Group 8 and 9 received AST along with naringin at 50 & 15 and 50 & 30 mg/kg, respectively. Group 10 animals served as normal control. AST was administered 30 min after oral dose of naringin. Blood samples (~200 μL) have been collected through retro-orbital puncture under light ether anesthesia just prior to and at 1^st^, 2^nd^ and 4^th^ h after AST administration in vials containing disodium EDTA as an anticoagulant. Plasma was separated and divided into two aliquots. One aliquot was immediately used to estimate total cholesterol and triglycerides levels using commercially available kits at 510 nm. The other aliquot was stored at -20 ± 5 °C until analysis for the quantification of AST.

The percentage variation of total cholesterol and triglycerides were calculated for each group using the following formula:



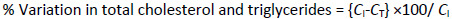



where, *C*_I_ is the concentration of disease control and *C*_T_ is the concentration of AST at 1^st^, 2^nd^ and 4^th^ hour.

### Quantitation of AST in rat plasma samples by RP-HPLC

Quantitation of AST in rat plasma samples were carried out by RP-HPLC using earlier reported method with minor modifications [26]. HPLC (Shimadzu, Japan) is equipped with LC-20 AT VP system controller, LC-20 AT pump, SPD-20A UV detector and a Phenomenex-C18 column (250 × 2.6 mm, 5 μm). The data were acquired and processed using LC solutions (version 3.1) software.

To an aliquot of 100 μL of rat plasma, 200 μL of methanol was added. The mixture was vortexed for 20 min followed by centrifugation at 3000 rpm for 10 min at 4 °C. The organic layer was separated and filtered through a 0.2 μm cellulose acetate filter. The chromatographic resolution of AST was achieved by using the isocratic mobile phase consists of acetonitrile : 0.05M sodium phosphate buffer (pH 4; 65:35, v/v) delivered at a flow rate of 1.0 mL/min and the eluent was monitored by UV detector set at 236 nm. An aliquot of 20 μL of organic layer was injected onto column and corresponding peak areas were noted. The calibration curve (y= 86.89x + 3832) was found to be linear from 100 to 2000 ng/mL (*r*^2^=0.9987).

### Statistical analysis

All the values were expressed as a Mean ± SEM. Results were analyzed statistically using one-way analysis of variance (ANOVA) following Dunnett test. Values of p<0.05 were considered statistically significant.

## Results

### Effect of naringin on AST in tyloxapol induced hyperlipidemia

#### Effect on total cholesterol

Administration of tyloxapol has significantly increased the total cholesterol after 30 h. It was observed that cholesterol levels were raised by 2.5 folds. The administration of AST alone at two test dose levels caused a statistical significant (p<0.001) dose dependent decrease in total cholesterol levels with maximum reduction observed at 2^nd^ h after drug administration (Table 1). Among the two test dose levels, AST with 50 mg/kg has produced maximum protection, with % reduction of total cholesterol was 19.09, whereas a percentage reduction of total cholesterol in animals received 25 mg/kg was 12.70 (Table 1). The effect of naringin alone on hyperlipidemia was not significant. The percent reduction in total cholesterol was 1.89 and 4.93 with 15 and 30 mg/kg, respectively at 2 h post administration of naringin (Table 1). The co-administration of naringin (15 and 30 mg/kg) with AST at two test dose levels (25 and 50 mg/kg) has produced significant reduction in total cholesterol levels and is quantitatively high at 2nd and 4th h after treatment when compared to AST alone treated animals (Table 1). However, the maximum percent reduction was noted at 2^nd^ h. The reduction in total cholesterol with AST (50 mg/kg) when co-administered with naringin (15 and 30 mg/kg) was 33.00 and 41.46 %, respectively where the total cholesterol is reduced by 19.09 % in animals received 50 mg/kg AST alone. Similarly, the reduction in total cholesterol was 24.02 and 31.30 % in animals received the AST (25 mg/kg) along with naringin (15 and 30 mg/kg). The reduction in total cholesterol is 12.70 % when treated with AST (25 mg/kg) alone (Table 1).

#### Effect on triglycerides

Administration of tyloxapol has increased the triglycerides levels to 9 to 9.5-folds from normal levels. The increased triglycerides levels were in the range of 650 to 695 mg/dL, when compared to initial levels in the range of 60-80 mg/dL. There is no significant fall in triglyceride levels in control rats during the course of experiment. The effect of naringin alone on triglyceride levels was not significant and the fall in the triglyceride levels in naringin treated rats is 2.04 and 4.08 % with 15 and 30 mg/kg doses, respectively, when compared to control rats (Table 2). The administration of AST alone has produced a significant dose dependent fall in triglyceride levels. The maximum reduction was observed at 2^nd^ h of the experiment with percentage reduction of 13.40 and 17.15 at dose levels of 25 and 50 mg/kg, respectively (Table 2). The co-administration of naringin (15 and 30 mg/kg) along with AST at two test dose levels statistically significant increase in the fall in triglyceride levels when compared to AST alone treated animals. The percent reduction in animals treated with AST along with naringin (25 + 15; 25 + 30 mg/kg) was 20.03 and 26.77, respectively. Whereas the percent reduction was 28.10 and 38.98 with test dose of AST and naringin at 50 + 15 and 50 + 30 mg/kg, respectively after 2 h post-treatment. The results pertaining to effect on triglyceride levels were shown in Table 2.

#### Quantitation of AST in rat plasma samples by RP-HPLC

**Figure 1.**
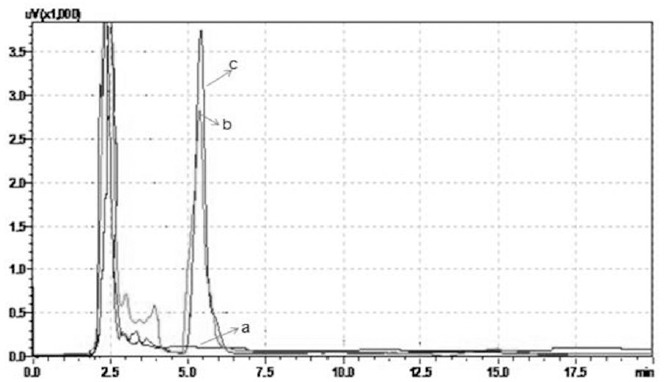
HPLC chromatograms of (**a**) rat blank plasma (**b**) rat plasma spiked with AST (300 ng/mL) and (**c**) 1 h *in vivo* rat plasma sample obtained after oral administration of AST at 50 mg/kg along with naringin 15 mg/kg (Group 9).

The plasma concentration of AST was determined in hyperlipidemic rats at 1, 2 and 4th h after oral administration of AST alone and AST along with naringin. HPLC chromatograms of (a) rat blank plasma (b) rat plasma spiked with AST (300 ng/mL) and (c) 1 h in vivo rat plasma sample obtained after oral administration of AST at 50 mg/kg along with naringin 15 mg/kg (Group 9) were shown in Figure 1. The maximum plasma concentration of AST was observed at 2nd h after oral administration of AST and it was found to be 43.33 and 198.92 ng/mL at dose levels of 25 and 50 mg/kg, respectively in AST alone treated animals. Table 3 the plasma concentration of AST. The concentration of AST is increased proportionally with increase in naringin dose. The plasma concentration of AST has increased with the co-administration of naringin at dose levels of 25 + 15, 25 + 30, 50 + 15 and 50 + 30 mg/kg was found to be 288.5, 663.81, 793.03 and 1233.72 ng/mL, respectively.

## Discussion

A bioenhancer is an agent capable of enhancing the bioavailability/bioefficacy of a particular drug with which it is co-administered. Naringin is a flavanone glycoside obtained from citrus fruits. Naringin possess diverse pharmacological properties. Previously, it was reported that supplementation with naringin for 3 weeks did not exhibit a hypolipidemic effect, however it reported to show beneficial effects of lowering hepatic cholesterol biosynthesis and levels of plasma lipids when supplemented for 6 weeks in a high fructose and high cholesterol fed rat model [27]. Naringin is known to enhancement of bioavailability of various structurally and therapeutically diverse drugs (verapamil, diltiazem, paclitaxel, tamoxifen) in rats by inhibition of CYP mediated metabolism and/or P-gp mediated permeability [19-22]. AST is an antihyperlipidemic agent belongs to statin family. It acts by blocking the enzyme responsible for cholesterol biosynthesis known as HMG Co-A reductase. AST is a potent dyslipidemic agent having significant use in lowering the blood circulation lipids such as low density lipoprotein (LDL), total-cholesterol (TC), triglycerides (TG) and apolipoproteins. AST is metabolized by CYP3A4. In the present study, the effect of naringin on antihyperlipidemic properties of AST was investigated with co-administration of naringin in tyloxapol induced hyperlipidemia in Wistar rats. The observed activity was correlated with plasma concentration of AST.

## Conclusions

The findings of the present study suggest that increased in plasma concentration of AST may be due to inhibition of CYPs and P-gp by naringin, which may be responsible for higher percent reduction in both cholesterol and triglycerides levels by AST. The treatment of hyperlipidemic patients with AST along with naringin rich diet may potentiate the therapeutic efficacy of AST and could be exploited to achieve better therapeutic control and patient compliance.

## Figures and Tables

**Table 1.** Effect of naringin on atorvastatin (AST) on total cholesterol levels induced by tyloxapol in male Wistar rats

Group	Treatment	Dose (mg/kg)	Plasma concentration of total cholesterol (mg/dL)			
			0 min	1 h	2 h	4 h
I	Disease control	-	132.2 ± 3.07	127.8 ± 2.28	137.8 ± 2.27	124.0 ± 2.44
II	Naringin	15	132.2 ± 1.99	126.5 ± 2.94 (1.02)	135.2 ± 2.35 (1.89)	122.2 ± 2.30 (1.45)
III	Naringin	30	129.7 ± 2.26	125.3 ± 2.10 (1.96)	131.0 ± 2.78 (4.93)	119.2 ± 2.02 (3.87)
IV	AST	25	135.3 ± 3.08	118.8 ± 2.72 (7.04)	120.3 ± 2.92*** (12.70)	110.0 ± 2.01*** (11.29)
V	AST + naringin	25 + 15	134.2 ± 2.34	111.3 ± 2.51*** (12.91)	104.7 ± 2.07*** (24.02)	98.83 ± 2.70*** (20.30)
VI	AST + naringin	25 + 30	132.0 ± 3.07	108.8 ± 3.19*** (14.87)	94.67 ± 2.60*** (31.30)	94.17 ± 2.71*** (24.06)
VII	AST	50	132.8 ± 2.33	115.7 ± 2.96* (9.47)	111.5 ± 2.95*** (19.09)	106.2 ± 2.30*** (14.35)
VIII	AST + naringin	50 + 15	134.7 ± 2.98	110.7 ± 2.81*** (13.38)	92.33 ± 2.02*** (33.00)	95.00 ± 2.60*** (23.39)
IX	AST + naringin	50 + 30	135.8 ± 2.41	106.2 ± 3.04*** (16.90)	80.67 ± 1.94*** (41.46)	88.17 ± 2.41*** (28.90)
X	Normal	-	54.0 ± 1.91	54.30 ± 1.82	54.50 ± 1.45	53.50 ± 1.94

Values are expressed as mean ± SEM,

**p<0.01,

***p<0.001; n=6

Figures in parenthesis indicate the percentage decrease in total cholesterol levels

**Table 2.** Effect of naringin on atorvastatin (AST) on triglyceride levels induced by tyloxapol in male Wistar rats

Group	Treatment	Dose (mg/kg)	Plasma concentration of total triglycerides (mg/dL)			
			0 min	1 h	2 h	4 h
I	Disease control	-	652.5 ± 2.74	670.8 ± 1.83	694.0 ± 3.07	662.2 ± 2.63
II	Naringin	15	666.8 ± 2.30	661.3 ± 2.27 (1.42)	674.2 ± 2.27*** (2.85)	648.7 ± 2.45** (2.04)
III	Naringin	30	663.8 ± 1.90	650.5 ± 2.47*** (3.03)	659.8 ± 2.35*** (4.93)	635.2 ± 2.02*** (4.08)
IV	AST	25	664.0 ± 2.87	640.2 ± 2.25*** (4.56)	601.0 ± 2.49*** (13.40)	591.2 ± 2.27*** (10.72)
V	AST + naringin	25 + 15	660.3 ± 2.36	615.0 ± 2.78*** (8.32)	555.0 ± 1.93*** (20.03)	567.7 ± 2.99*** (14.27)
VI	AST + naringin	25 + 30	668.0 ± 2.54	594.2 ± 2.89*** (11.4)	508.2 ± 2.75*** (26.77)	522.0 ± 2.12*** (21.17)
VII	AST	50	660.5 ± 1.97	630.5 ± 2.71*** (6.01)	575.0 ± 1.75*** (17.15)	575.7 ± 2.10*** (13.06)
VIII	AST + naringin	50 + 15	670.2 ± 2.86	589.8 ± 3.16*** (12.08)	499.0 ± 2.06*** (28.10)	522.3 ± 1.76*** (21.13)
IX	AST + naringin	50 + 30	670.3 ± 2.43	563.8 ± 2.64*** (15.95)	423.5 ± 2.32*** (38.98)	482.3 ± 2.43*** (27.17)
X	Normal	-	71.50 ± 2.46	73.50 ± 1.74	74.00 ± 1.62	73.17 ± 2.02

Values are expressed as mean ± SEM,

**p<0.01,

***p<0.001; n=6

Figures in parenthesis indicate the percentage decrease in total triglycreide levels

**Table 3.** Effect of naringin on pharmacokinetic profile of atorvastatin (AST) in tyloxapol induced hyperlipidemia male Wistar rats

Group	Treatment	Dose (mg/kg)	Concentration (ng/mL)		
			1^st^ hour	2^nd^ hour	4^th^ hour
IV	AST	25	20.53	43.33	39.48
V	AST + naringin	25 + 15	144.7	288.5	226.3
VI	AST + naringin	25 + 30	484.8	663.8	596.4
VII	AST	50	82.86	198.9	159.2
VIII	AST + naringin	50 + 15	301.9	793.0	650.7
IX	AST + naringin	50 + 30	508.9	1233.7	951.5
